# Propargylic substitution reactions with various nucleophilic compounds using efficient and recyclable mesoporous silica spheres embedded with FeCo/graphitic shell nanocrystals

**DOI:** 10.1186/1556-276X-10-2

**Published:** 2015-01-23

**Authors:** Seongwan Jang, A Young Kim, Won Seok Seo, Kang Hyun Park

**Affiliations:** Department of Chemistry and Chemistry Institute for Functional Materials, Pusan National University, Busan, 609-735 South Korea; Department of Chemistry, Sogang University, Seoul, 121-742 South Korea

**Keywords:** Recyclable, Magnetic, FeCo/GC, Propargylic substitution, Phosphomolybdic acid

## Abstract

Phosphomolybdic acid (PMA, H_3_PMo_12_O_40_) functioned as a catalyst for reactions of secondary propargylic alcohols and nucleophiles. Highly stable and magnetically recyclable mesoporous silica spheres (MMS) embedded with FeCo-graphitic carbon shell nanocrystals (FeCo/GC@MSS) were fabricated by a modified Stöber process and chemical vapor deposition (CVD) method. The FeCo/GC@MSS were loaded with phosphomolybdic acid (PMA@FeCo/GC@MSS), and their catalytic activity was investigated. Propargylic reactions of 1,3-diphenyl-2-propyn-1-ol with a wide range of nucleophiles bearing activating substituents were catalyzed under mild conditions. It was found that the MMS possess mesoporosities and have enough inner space to load FeCo and phosphomolybdic acid. The FeCo/GC@MSS were found to be chemically stable against acid etching and oxidation. This suggests that the nanocrystals can be used as a support for an acid catalyst. Moreover, the magnetic property of the nanocrystals enabled the facile separation of catalysts from the products.

## Background

Electrophilic attack on aromatic carbons is a useful method for functionalizing aromatic compounds [1–3]. Electrophilic aromatic substitution is an organic reaction, in which an electrophile replaces an atom (usually hydrogen) appended to an aromatic system. Among these reactions, the most important are the nitration, halogenation, sulfonation, and acylation reactions of aromatic compounds. Propargylic substitution reactions have been intensively studied in recent years. In these reactions, activated and inactivated propargyl alcohols, propargyl acetates, and/or propargyl esters react with alcohols, thiols, amines, and other molecules that have C-nucleophiles and heteroatom-centered nucleophiles [4, 5].

Heteropoly acids have been the focus of extensive research in organic synthesis due to their high catalytic activity, ease of control, and low cost [6]. Among the various heteropoly acids, phosphomolybdic acid (PMA, H_3_PMo_12_O_40_) is one of the least expensive commercially available solid acids [7–11]. PMA not only enhances the activity of selected catalysts but also shows self-catalytic activity in various organic reactions [11–15]. However, the recovery and reuse of PMA still remains a challenge. Our efforts toward green chemistry have led to the development of new synthetic methodologies.

**Figure 1:**
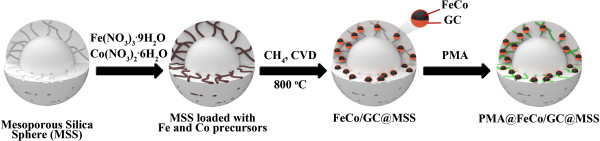
**Schematic diagram for the preparation of a PMA@FeCo/GC@MSS.**

Recently, we reported a simple one-step chemical vapor deposition (CVD) method to synthesize highly stable and magnetically recyclable mesoporous silica spheres (MSS) embedded with FeCo-graphitic carbon shell nanocrystals (FeCo/GC@MSS) [16]. The schematic strategy for the preparation of FeCo/GC@MSS and PMA loading is illustrated in Figure 1. In continuation of our previous studies on the catalytic properties of PMA@FeCo/GC@MSS as an inexpensive and eco-friendly reagent, we disclose O-nucleophilic substitution reactions of aryl propargyl methanol. We have shown that silica-supported PMA works as an excellent recyclable solid reagent in catalyzing propargylic substitution reactions. To investigate PMA@FeCo/GC@MSS as a solid reagent system, we disclose herein propargylic substitution reactions of 1,3-diphenyl-2-propyn-1-ol with various nucleophilic compounds in acetonitrile.

## Methods

### General remarks

Iron(III) nitrate nonahydrate (Fe(NO_3_)_3_ · 9H_2_O, 99.99%), cobalt(II) nitrate hexahydrate (Co(NO_3_)_2_ · 6H_2_O, 99.999%), and phosphomolybdic acid (PMA) hydrate (H_3_PMo_12_O_40_ · 24H_2_O, 99.99%) were purchased from Sigma-Aldrich, St. Louis, MO, USA. Tetraethoxysilane (TEOS, 98%, Sigma-Aldrich, St. Louis, MO, USA) and n-octadecyltrimethoxysilane (C_18_TMS, 85%) were purchased from TCI, Tokyo Japan. All chemicals were used as received without further purification.

### Synthesis of FeCo/GC@MSS, FeCo/GC, and FeCo@MSS nanocrystals

Mesoporous silica spheres composed of mesoporous shell and solid core (approximately 400 nm) were prepared by modifying the Stöber process [15]. We added 1.00 g of MSS with 0.22 g (0.52 mmol) of Fe(NO_3_)_3_ · 9H_2_O and 0.12 g (0.38 mmol) of Co(NO_3_)_2_ · 6H_2_O in 50 mL of methanol and then sonicated it for 1 h. The samples were then dried at 80°C and placed in a tube furnace and heated under H_2_ flow at 800°C. The samples were then subjected to a methane flow of 500 cm^3^/min^−1^ for 5 min. After cooling, the samples were washed with ethanol and collected by centrifugation. To obtain the FeCo/GC nanocrystals, the samples were etched with 15% hydrogen fluoride (HF) in H_2_O (75%) and ethanol (10%) to dissolve the silica. The procedure for the synthesis of FeCo@MSS was similar to that of FeCo/GC@MSS, except that the methane flow at 800°C for 5 min was replaced with H_2_ flow at 800°C.

### Synthesis of (PMA@FeCo/GC@MSS) nanocrystals

To prepare PMA@FeCo/GC@MSS, 0.82 g of FeCo/GC@MSS nanoparticles were added slowly to a solution of H_3_PMo_12_O_40_ · 24H_2_O (0.09 g, 0.05 mmol) in methanol (10 mL). The mixture was stirred at room temperature for 6 h, and the solvent was removed under reduced pressure to obtain 10 wt% PMA in SiO_2_ (a greenish-black powder).

### Characterization

The morphology and structure of the samples were investigated by transmission electron microscopy (TEM) (JEOL JEM-2100 F, Akishima-shi, Japan operated at 200 KV) with selected area electron diffraction patterns and energy dispersive analyses of X-ray emission. The samples for the TEM analyses were prepared by adding the diluted sample to ethanol drop-wise on a 300-mesh carbon support copper grid (Ted Pella, Inc., Redding, CA, USA). Powdered X-ray diffraction (XRD) patterns were collected on a Rigaku Miniflex II (4.5 KW) diffractometer (Rigaku Corporation, Shibuya-ku, Tokyo) using Cu-Kα radiation at 30 kV and 15 mA. The magnetic measurements were carried out on a superconducting quantum interference device (SQUID) magnetometer (Quantum Design MPMS SQUID-VSM, Quantum Design, San Diego, USA). The Brunauer-Emmett-Teller (BET)-specific surface areas and porosity of the samples were evaluated on the basis of nitrogen adsorption isotherms using a BELSORP-max instrument (BELSORP-max, Nippon Bell, Japan).

### Propargylic substitution reactions of PMA@MSS@FeCo/GC nanocatalysts

Propargylic substitution reactions of 1,3-diphenyl-2-propyn-1-ol were carried out in a 10-mL glass vial. PMA@MSS@FeCo/GC nanocatalysts (0.05 mol%), 1,3-diphenyl-2-propyn-1-ol (0.19 ml, 1.0 mmol), phenol (0.113 g, 1.2 mmol), and acetonitrile (5.0 mL) were added, and the mixture was stirred for 30 min at 323 K. Following the reaction, the nanoparticles were separated from the solution with a magnet. The reaction products were analyzed using a ^1^H NMR Varian Mercury Plus spectrometer (300 MHz) (Varian, Inc., Palo Alto, CA, USA). Chemical shift values were recorded in parts per million relative to tetramethylsilane as an internal standard unless otherwise indicated, and the coupling constants were reported in Hertz.

## Results and discussion

### Synthesis and structural characterization

The major steps involved in the synthesis of PMA@FeCo/GC@MSS are highlighted in Figure 1. We prepared FeCo/GC@MSS as a light gray powder by modifying the Stöber process [15] and CVD method. The MSS were then used as templates for loading FeCo/GC and PMA. A 0.9 mmol of metal precursors, Fe(NO_3_)_3_ · 9H_2_O and Co(NO_3_)_2_ · 6H_2_O, at a 58:42 molar ratio were loaded onto 1.0 g of the MSS by impregnation in methanol solutions, followed by solvent removal under reduced pressure. To deposit carbon on to the FeCo nanocrystals formed in the MSS, the metal-loaded MSS was heated to 800°C under H_2_ and then subjected to methane CVD. Once the MSS were cooled to room temperature, any metal impurities were removed by washing with a 10% aqueous HCl. When loading PMA on FeCo/GC@MSS (10 wt% of PMA in SiO_2_), FeCo/GC@MSS was added to PMA dissolved in methanol and then sonicated for 5 min. This was followed by stirring for 6 h at room temperature and solvent removal under reduced pressure to afford PMA@FeCo/GC@MSS as a light greenish powder.

**Figure 2:**
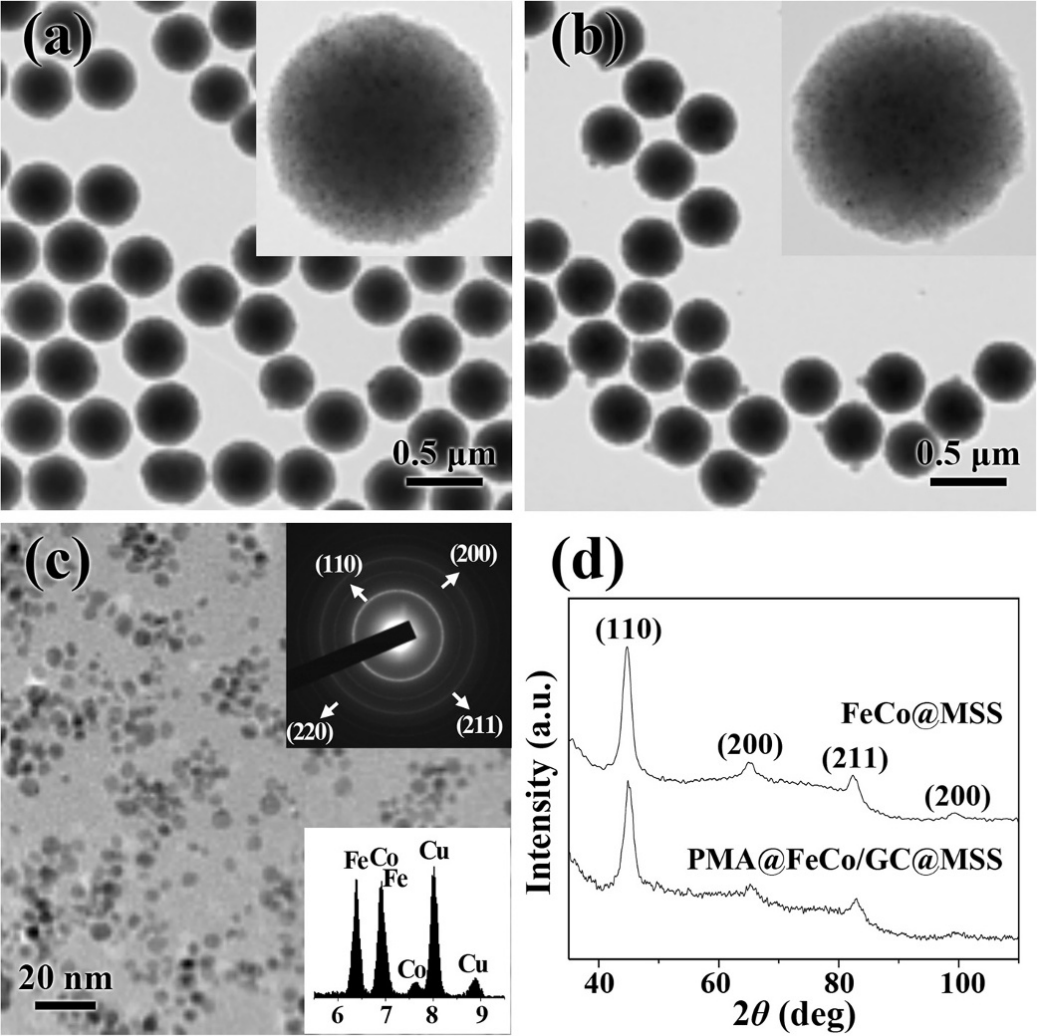
**Morphology and structure of FeCo/GC@MSS and PMA@FeCo/GC@MSS.** TEM images of **(a)** FeCo/GC@MSS and **(b)** PMA@FeCo/GC@MSS (Insets are higher magnification images.). **(c)** TEM image of FeCo/GC nanocrystals (Upper inset is the electron diffraction pattern. Lower inset is the EDX spectrum. Copper is from the TEM grids.) **(d)** X-ray diffraction patterns.

The representatives FeCo/GC@MSS and PMA@FeCo/GC@MSS are compiled in Figures 2a and 1b, respectively. The TEM images of an FeCo/GC@MSS in the inset of Figure 2a and PMA@FeCo/GC@MSS in the inset of Figure 2b clearly show the FeCo/GC nanocrystals embedded in the MSS. After treatment of the FeCo/GC@MSS with HF to dissolve the silica, we obtained FeCo/GC nanocrystals with an average diameter of 5.6 ± 1.0 nm, as shown in Figure 2c. Energy dispersive X-ray (EDX) spectrum of the nanocrystals (lower inset of Figure 2c) shows a Fe/Co ratio of 50:50, which is a slightly higher Co content than the Fe/Co ratio of precursors. The electron diffraction (upper inset of Figure 2c) and XRD patterns (Figure 2d) were used to observe the crystal structure of FeCo. The crystal structure was identified as a body-centered-cubic (bcc). The crystallite size was determined for the (110) reflection of the XRD data (Figure 2d) by using the Debye-Scherrer equation [17]. It was found to be 5.3 nm, indicating a single-crystalline and spherical nature for the individual FeCo/GC nanocrystals.

**Figure 3:**
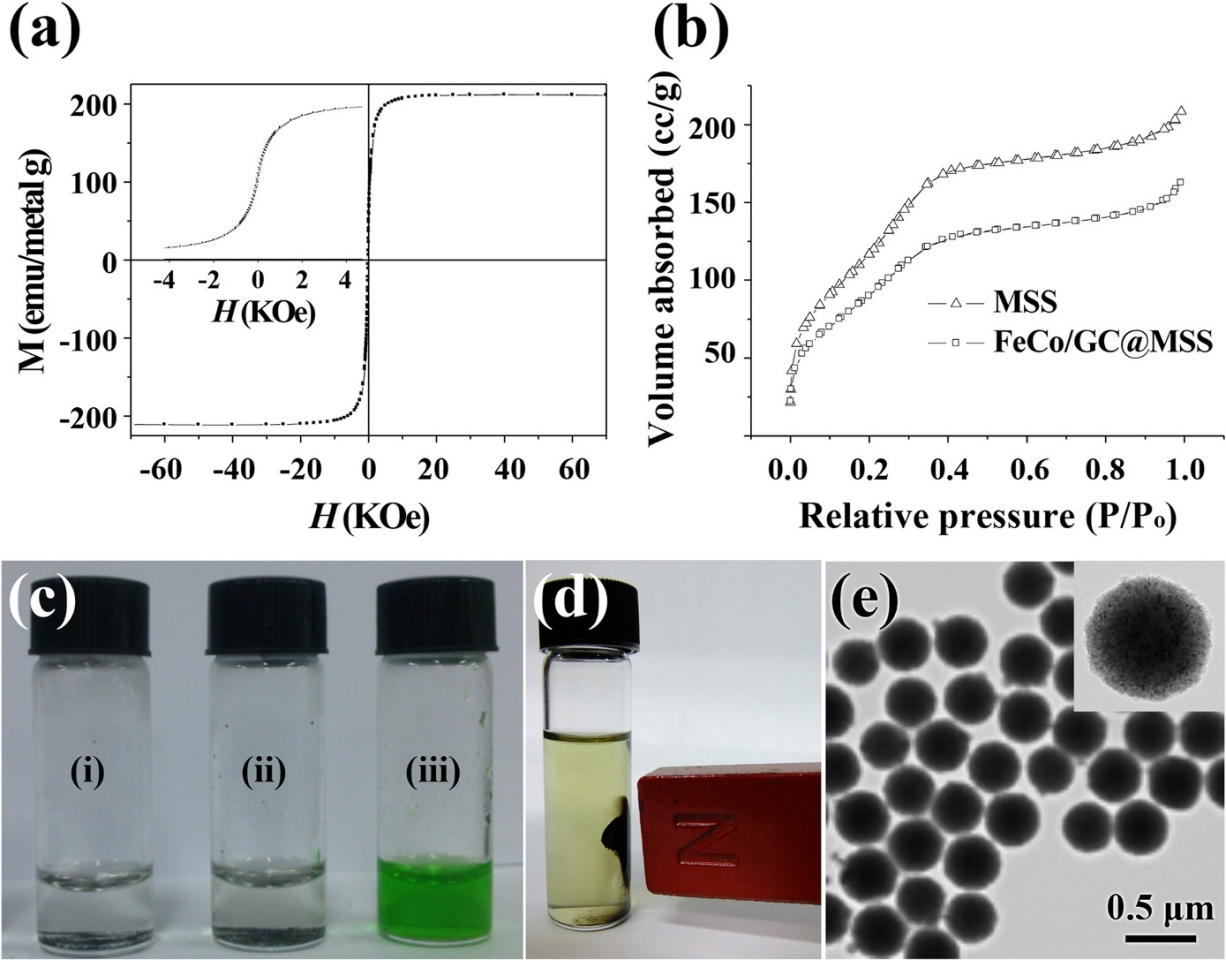
**Suitability of FeCo/GC@MSS for use in the reaction system. (a)** Field-dependent magnetization hysteresis of FeCo/GC@MSS at 300 K. **(b)** Nitrogen adsorption/desorption isotherm of MSS and FeCo/GC@MSS. **(c)** Photographs of 35% HCl solutions of (i, ii) FeCo/GC@MSS stored over a monitoring period of 2 months in air (i) and water (ii) and (iii) as-prepared FeCo@MSS. **(d)** A photograph of recycled PMA@FeCo/GC@MSS in acetonitrile in the presence of an external magnet. **(e)** TEM image of the PMA@FeCo/GC@MSS after the five sequential catalytic cycles.

The magnetic properties of the FeCo/GC@MSS were investigated by SQUID magnetometry. Figure 3a shows the magnetization hysteresis curves for the FeCo/GC@MSS sample. The saturation magnetization value was obtained as high as 211 emu/metal g. The BET surface area, total pore volume, and calculated average pore volume of the FeCo/GC@MSS were calculated to be 315.8 m^2^/g, 0.239 cm^3^/g, and 2.9 nm, respectively. The values are slightly smaller than those for the MSS (343.8 m^2^/g, 0.312 cm^3^/g, and 3.1 nm, respectively) due to the embedment of the approximately 5.6 nm FeCo/GC nanocrystals. Nevertheless, the N_2_ adsorption isotherms (Figure 3b) of the FeCo/GC@MSS show type IV curves, which is typical for mesoporous silica [18]. This indicates that the pores of FeCo/GC@MSS might have a sufficiently large inner space to allow high performance in catalytic reactions.The FeCo/GC@MSS also shows long-term chemical stability (Figure 3c). The samples were stored for 2 months in air and water and then etched with HCl. Both samples still exhibited excellent stability against HCl etching even after the air or water storage. However, the FeCo@MSS sample solutions prepared for comparison purpose turned green after the addition of HCl due to Fe and Co etching. This infers that FeCo/GC@MSS is still stable against oxidation for a long time in air or water due to the robustness of the single-layered graphitic shell.

### Propargylic substitution reactions of 1,3-diphenyl-2-propyn-1-ol with PMA@FeCo/GC@MSS

The catalytic activity of PMA@FeCo/GC@MSS was assessed by studying the propargylic substitution reactions of 1,3-diphenyl-2-propyn-1-ol with phenol. Srihari et al. reported efficient solvents in propargylic substitution reactions using PMA catalysts [14]. Hydrophilic solvents such as water and PEG 400 showed low functional group conversion, whereas hydrophobic solvents such as acetonitrile, dichloromethane, and dichloroethane showed high conversion. Therefore, acetonitrile was selected as the solvent for investigating the catalytic activity of PMA@FeCo/GC@MSS. To optimize the reaction conditions, the amount of catalyst and reaction time were varied over a series of reactions. In general, it was found that increasing the amount of catalyst, reaction temperature, and reaction time were effective means of increasing conversion (Table 1, entries 1 to 7). Under common conditions at 323 K, conversion approaches 100% with a reaction time of 30 min and when 0.05 mol% PMA is present (Table 1, entry 5). The 0.05 mol% PMA@FeCo/GC@MSS was used in subsequent reactions owing to a reasonable turnover frequency (TOF) and conversion. For comparison, the corresponding homogeneous reaction was also carried out, under the same conditions described above (Table 1, entry 8). Compared with pure PMA, PMA@FeCo/GC@MSS showed similar catalytic activity. As expected, the MSS and FeCo/GC@MSS did not exhibit any catalytic activity (Table 1, entries 9, 10). Therefore, the observed catalytic conversion by PMA@FeCo/GC@MSS is attributed to the incorporated PMA clusters. As shown in Figure 3d, the resulting PMA@FeCo/GC@MSS catalyst is easily recycled by magnetic separation whilst keeping its high catalytic activity in propargylic substitution reactions of 1,3-diphenyl-2-propyn-1-ol (Table 1, entries 11 to 14). As shown in Figure 3e, the morphology of the catalyst remained the same after five consecutive catalytic cycles.

**Table 1:** **Propargylic substitution reactions of 1,3-diphenyl-2-propyn-1-ol with phenol using PMA@MSS@FeCo/GC** ^**a**^


Entry	Catalyst (PMA mol%)	Time (min)	Temp. (°C)	Conv. (%) ^b^	TOF (−h)
1	0.03 mol%	30	50	79	5,267
	PMA@FeCo/GC@MSS				
2	0.05 mol%	5	50	81	19,440
	PMA@FeCo/GC@MSS				
3	0.05 mol%	10	50	94	11,280
	PMA@FeCo/GC@MSS				
4	0.05 mol%	20	50	97	5,820
	PMA@FeCo/GC@MSS				
5	0.05 mol%	30	50	100	4,000
	PMA@FeCo/GC@MSS				
6	0.1 mol%	30	25	93	1,860
	PMA@FeCo/GC@MSS				
7	0.1 mol%	30	50	100	2,000
	PMA@FeCo/GC@MSS				
8	0.05 mol% PMA	30	50	95	3,800
9	FeCo/GC@MSS^c^	30	50	0	-
10	SiO_2_^c^	30	50	0	-
11	Recovered from number 5	30	50	100	4,000
12	Recovered from number 11	30	50	100	4,000
13	Recovered from number 12	30	50	100	4,000
14	Recovered from number 13	30	50	100	4,000

^a^Reaction conditions: 1,3-diphenyl-2-propyn-1-ol (0.19 ml, 1.0 mmol), phenol (1.2 mmol), and acetonitrile (5.0 mL). ^b^Determined by ^1^H NMR spectroscopy. Yields are based on the amount of propargylic alcohol. ^C^Amount of compound based on calculated content in 0.05 mol% PMA@FeCo/GC@MSS, respectively.

We applied the optimized reaction procedure to the reactions of 1,3-diphenylprop-2-yn-1-ol with various nucleophiles. Electron-donating substituents on the aromatic ring located near the nucleophiles were found to enhance the reactivity (Table 2, entries 1 to 4). For all the nucleophiles, substitution involved regioselective attack by the aromatic carbon with the highest electron density. Furthermore, allyl trimethyl silane underwent C-nucleophilic substitution under these reaction conditions (Table 2, entry 5). Conversely, 2-propene-1-ol, 2-propyn-1-ol, cyclopentanamine, and thiophenol afforded ether and thioether exclusively without C-nucleophilic-substituted products (Table 2, entries 6 to 9). This may be attributed to the more nucleophilic character of a heteroatom than carbon. Conversion of 2-propene-1-ol was higher than 2-propyn-1-ol owing to the difference in the inductive effects between the sp^2^ and sp character. Unfortunately, these nucleophiles showed poor reactivity in this reaction.

**Table 2:** **Propargylic substitution reactions of 1,3-diphenyl-2-propyn-1-ol with various nucleophilic compounds using PMA@MSS@FeCo/GC** ^**a**^


Entry	Substrate	Product	Conversion (%) ^b^
1			100
2			99
3			94
4			75
5			67
6			93
7			76
8			31
9			79

^a^Reaction conditions: 1,3-diphenyl-2-propyn-1-ol (0.19 ml, 1.0 mmol), nucleophile (1.2 mmol), acetonitrile (5.0 mL), catalyst (0.05 mol%), and time ( 30 min). ^b^Determined by ^1^H NMR spectroscopy. Yields are based on the amount of propargylic alcohol.

**Figure 4:**
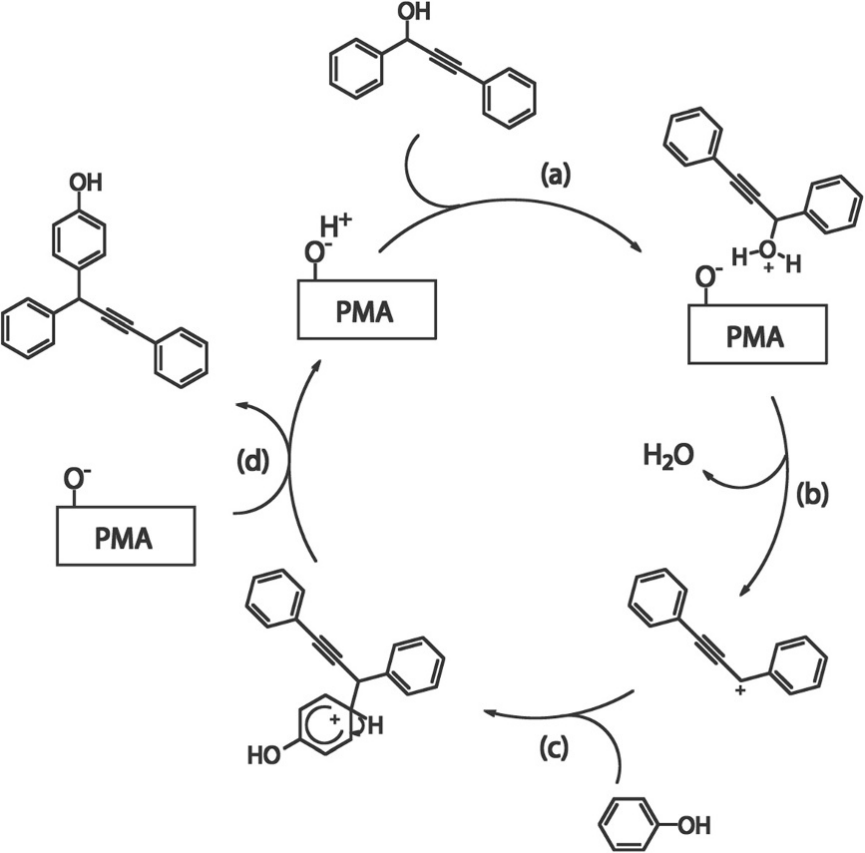
**Plausible mechanism for the PMA@FeCo/GC@MSS-catalyzed propargylic substitution reactions. (a)** Protonation of hydroxyl group of propargylic alcohol. **(b)** Generation of propargylic carbenium ion by dehydration. **(c)** Donation of electron from electron-rich arene **(d)** removal of proton from the previous intermediate.

Mechanisms of propargylation reactions have been proposed by several groups [19, 20]. The mechanism of the propargylic substitution reactions for PMA@FeCo/GC@MSS may follow these reported mechanisms. This reaction follows the S_N_1 mechanism and the propargyl cations act as reactive intermediates in the reaction. First, the hydroxyl group of propargylic alcohol is protonated by the H^+^ active site (Figure 4a), and it then generates propargylic carbenium ion through dehydration (Figure 4b). Next, an electron is donated from an electron-rich arene (such as phenol) to the carbenium compound (Figure 4c). In this step, the aromatic ring stabilizes the cation by resonance effects. Finally, the product is obtained by the removal of a proton from the previous intermediate, and this proton regenerates the H^+^-active site of PMA (Figure 4d).

## Conclusions

In summary, we have successfully prepared MSS embedded with FeCo/GC nanocrystals through a simple one-step CVD process. This superparamagnetic FeCo/GC@MSS showed high saturation magnetization and superior chemical stability against acid etching and oxidation. PMA-loaded FeCo/GC@MSS worked as a green catalyst for propargylic substitution reactions of various aromatic compounds with 1,3-diphenylprop-2-yn-1-ol. The catalyst can be easily separated and reused at least five times without any appreciable loss in its catalytic efficiency, thereby showing great potential for large-scale applications. The results indicate that such materials can be used as catalysts in organic reactions.
